# A qualitative assessment on the acceptability of providing cash transfers and social health insurance for tuberculosis-affected families in Ho Chi Minh City, Vietnam

**DOI:** 10.1371/journal.pgph.0002439

**Published:** 2023-12-06

**Authors:** Rachel Forse, Thanh Thi Nguyen, Thu Dam, Luan Nguyen Quang Vo, Andrew James Codlin, Maxine Caws, Ha Dang Thi Minh, Lan Huu Nguyen, Hoa Binh Nguyen, Nhung Viet Nguyen, Knut Lönnroth, Kristi Sidney Annerstedt

**Affiliations:** 1 Friends for International TB Relief, Hanoi, Vietnam; 2 Department of Global Public Health, Karolinska Institutet, WHO Collaboration Centre on Tuberculosis and Social Medicine, Stockholm, Sweden; 3 Department of Clinical Sciences, Liverpool School of Tropical Medicine, Liverpool, United Kingdom; 4 Pham Ngoc Thach Hospital, Ho Chi Minh City, Vietnam; 5 National Lung Hospital/National TB Control Programme, Hanoi, Vietnam; 6 University of Medicine and Pharmacy, Vietnam National University, Hanoi, Vietnam; BRAC University, BANGLADESH

## Abstract

To achieve the Sustainable Development Goal’s targets of universal health coverage (UHC) and poverty reduction, interventions are required that strengthen and harmonize both UHC and social protection. Vietnam is committed to achieving financial protection and over 90% of the general population has enrolled in its social health insurance (SHI) scheme. However, an estimated 63% of tuberculosis (TB)-affected households in Vietnam still face catastrophic costs and little is known about the optimal strategies to mitigate the costs of TB care for vulnerable families. This study assessed the acceptability of a social protection package containing cash transfers and SHI using individual interviews (n = 19) and focus group discussions (n = 3 groups). Interviews were analyzed through framework analysis. The study’s main finding indicated that both conditional and unconditional cash transfers paired with SHI were acceptable, across six dimensions of acceptability. Cash transfers were considered beneficial for mitigating out-of-pocket expenditure, increasing TB treatment adherence, and improving mental health and general well-being, but the value provided was inadequate to fully alleviate the economic burden of the illness. The conditionality of the cash transfers was not viewed by participants as inappropriate, but it increased the workload of the TB program, which brought into question the feasibility of scale-up. SHI was viewed as a necessity by almost all participants, but people with TB questioned the quality of care received when utilizing it for auxiliary TB services. Access to multiple sources of social protection was deemed necessary to fully offset the costs of TB care. Additional research is needed to assess the impact of cash transfer interventions on health and economic outcomes in order to create an enabling policy environment for scale-up.

## Introduction

The World Health Organization (WHO) has set ambitious targets for ending the global TB epidemic by 2030, including a 90% reduction in TB incidence, a 95% reduction in TB mortality and the elimination of catastrophic costs due to TB [1]. This timeline and call to action aligns with the Sustainable Development Goals, particularly Health Goal No. 3.3, which calls for the end of the TB epidemic [2,3]. Subsequently, the Government of Vietnam created a plan to end TB by 2030 [4]. However, the challenges of achieving these targets remain significant, especially in light of the COVID-19 pandemic.

Vietnam ranks 10th among the world’s highest TB burden countries, with 172,000 people developing TB in 2020 and over 10,000 people dying from this preventable and curable disease [5]. Additionally, a national patient cost survey identified that 63% of TB-affected households experienced catastrophic costs, defined as having costs associated with TB care equivalent to more than 20% of their annual household income [6].

Cash transfers have emerged as a key social protection intervention to potentially improve health. They have been recommended as one means of expanding social protection in the WHO End TB strategy [7]. Establishing the optimal design of conditions placed on these transfers, if any, has been identified as a key research priority [8].

Cash transfers are the provision of money to vulnerable individuals; they can be distributed unconditionally or on the condition of a beneficiary participating a specific action, such as attendance at a medical appointment, adherence to a treatment plan, pick-up of nutritional supplements, periodic testing, or participation in vaccination [9,10]. Conditional cash transfers (CCTs) have been shown to generally increase adoption of targeted behaviors, increase use of health services and at times improve health outcomes [11]. Unconditional cash transfers (UCTs) have been shown to increase spending on household priorities, which often improves overall wellbeing, but direct impacts on health are not as evident [12].

A systematic review found seven ‘TB-specific’ interventions aiming to improve treatment outcomes through cash transfers in low- and middle-income countries (LMICs) [13]. Of these, six were at least partially conditioned on clinic attendance or treatment completion. Evidence from the systematic review suggests that cash transfer interventions can offset the costs of TB care in LMICs, and act as a poverty-reduction measure. Cash transfers may also improve clinical outcomes for people with TB, although this finding is based on a single randomized control trial [14]. To our knowledge, there are currently no published studies on cash transfers to mitigate the costs of TB in the Vietnamese context.

Any cash transfer intervention in Vietnam should integrate within the context of the national social security system. Since 1989, Vietnam has been progressing towards universal health coverage (UHC) through a government-provided Social Health Insurance (SHI) scheme. By 2019, over 90% of the population had been enrolled in SHI [15,16]. From the patient-perspective at the time of data collection of this study, most TB diagnostic evaluations, healthcare consultations and medications in the public sector were provided nominally free-of-charge through Vietnam’s National TB Programme (NTP) without requiring proof of SHI enrollment. Starting in mid-2022, after completion of data collection on this study, public health facilities at the secondary care level have been made eligible to receive reimbursements from SHI for TB treatment [17]. The effects of this change to the financing model for TB care in Vietnam have yet to be studied.

In parallel to progress made towards UHC, Vietnam’s NTP has begun to address the impoverishing effects of TB, including expansion of access to social protection. They created the Patients Support to Fight TB (PASTB) fund, a national financial risk reduction charity fund to purchase SHI for poor individuals without SHI coverage at the start of their TB treatment and to provide limited cash transfers [18]. Additionally, donor agencies and local partners are collaborating with the NTP to pilot the delivery of cash transfers and SHI enrollment and/or cash transfers for people with TB in Vietnam [19–21].

Acceptability is a key consideration for the design, delivery and evaluation of complex interventions to improve health outcomes [22,23]. For an intervention to be successful, it requires both healthcare providers and beneficiaries to find it acceptable [24]. If healthcare providers do not find an intervention to be acceptable, then it may not be delivered as designed, or the quality of service delivery may diminish [25]. For beneficiaries of an intervention, low acceptability can negatively impact perceived quality of care, uptake, adherence, and effectiveness [26,27].

However, in the scientific literature, a standardized definition of acceptability has not been applied, and often behavioral indicators, such as dropout rates, have been used as measurements of acceptability [28]. To systematically develop a common understanding of acceptability, Sekhon et al. developed a Theoretical Framework of Acceptability (TFA). This framework defines acceptability as a multi-dimensional construct across three temporal perspectives, and provides a clear conceptual definition [29].

As the provision of cash transfers and SHI for TB is being piloted and evaluated in Vietnam and other LMICs, the acceptability of these forms of social protection among health care providers and beneficiaries has not yet been rigorously evaluated, yet their acceptability is a necessary (albeit insufficient) condition for evaluating their effectiveness and scalability [29].

This study was embedded within an intervention that combined cash transfers with SHI for people with drug-susceptible TB in Vietnam, called the ‘cash transfer UHC+ intervention.’ It aimed to assess the multifaceted components of acceptability of this intervention, contribute to policy debates on conditionality of cash transfers and contextualize these findings within Vietnam and other LMICs.

## Materials and methods

We utilized the TFA to design a topic guide and structure the results of the study. This framework consists of seven distinct, yet interconnected, constructs of acceptability (See Table 1). It provided guidance to measure the extent to which healthcare providers and beneficiaries of the intervention considered it to be acceptable [29].

**Table 1:** Definitions of acceptability constructs*.

Affective attitude	How the participant feels about the intervention
Perceived effectiveness	The extent to which the intervention is perceived as likely to achieve its purpose
Ethicality	The extent to which the participant understands the intervention and how it works
Burden	The perceived amount of effort that is required to participate in the intervention
Intervention Coherence	The extent to which the participant understands the intervention
Opportunity Costs	The extent to which benefits, profits, or values must be given up to engage in the intervention
Self-efficacy	The participant’s confidence that they can perform the behavior(s) required to participate in the intervention

*Adapted from Sekhon M, Cartwright M, Francis JJ. Acceptability of healthcare interventions: An overview of reviews and development of a theoretical framework. BMC Health Services Research. 2017 Jan 26;17(1):88.

This exploratory qualitative study was conducted at two time points: June 2019 and June 2020. The first set of interviews (nine individual interviews, one focus group discussion [FGD]) was conducted prior to the cash transfer UHC+ intervention. The second set of interviews (10 individual interviews, two FGDs) was conducted after the intervention had completed enrollment. The methodological orientation of the study utilized policy and evaluation analysis, specifically Framework Analysis [30]. The C*onsolidated Criteria for Reporting Qualitative Research* (COREQ) checklist was used to ensure comprehensive reporting of the qualitative data [31] (See S1 File).

### Ethical approvals

Ethical approvals for the implementation were granted by the National Lung Hospital Institutional Review Board (114/19/CT-HĐKH-ĐĐ) in Hanoi, Vietnam. Study implementation was approved by the Ho Chi Minh City People’s Committee. We obtained written informed consent from all participants and anonymized all data prior to analysis.

### Study setting

Ho Chi Minh City is the largest city in Vietnam, with a registered population of approximately nine million people and close to 11 million people living in the metropolitan area [32]. Twenty interviews were conducted with participants in Ho Chi Minh City, while two were conducted with NTP staff in Hanoi.

### Cash transfer UHC+ intervention description

The cash transfer UHC+ intervention was designed to be similar to the forms of support provided by PASTB. It consisted of two components: the provision of either CCT or UCT to eligible people with drug-susceptible TB in four districts of Ho Chi Minh City and the purchase of SHI for all TB-affected households who did not have active coverage.

Eligible individuals had pulmonary drug-susceptible TB, and at the time of treatment initiation were identified as being economically vulnerable by holding government poverty registration, lacking SHI, and being unemployed or informally employed. Eligibility was assessed by healthcare providers working within District TB Units (DTUs). For both the CCT and UCT groups, the value of the cash transfer was 500,000 Vietnamese Dong (VND) (~21.50 USD, OANDA.com) per month and it was provided for six months. For UCTs, cash was distributed by program staff at the participant’s home or another convenient location, such as a coffee shop.

The conditions that needed to be met for beneficiaries to obtain the CCT included attending a monthly examination at a DTU, along with a demonstration that at least 85% of medications provided at the last appointment were no longer in the blister packs, as a proxy measure of treatment adherence. The cash transfer was provided by the DTU staff during a monthly appointment (See Table 2).

**Table 2:** Cash Transfer UHC+ intervention description.

Eligibility:	Cash transfer details:
**Clinical eligibility criteria (all needed to apply):** • Bacteriologically-confirmed, pulmonary TB • New or relapse case of TB • 18 years+ • Living in one of the four implementation districts • Obtaining treatment at a DTU in an implementation district **Socio-economic eligibility (any one needed to apply):** • Residing in a household officially declared a ‘poor household’ • Not enrolled in SHI at TB treatment initiation • Unemployed at TB treatment initiation Employed in the informal sector without labor protections	**CT value:** 500,000 VND (~21.50 USD) for 6 months **Payment frequency:** Monthly • UCT- Every 30 days after study enrollment • CCT- To be provided after meeting conditions at DTU **Mode of payment:** Cash **Location of payment:** • UCT at the beneficiary’s home, or other convenient location • CCT at the DTU
	**Conditions to receive CCT:** • Attend monthly appointment at DTU • Bring pill pack showing 85% of medication had been taken over the last month

**CCT**–Conditional cash transfers; **CT**–Cash transfers; **DTU**—District TB Unit; **SHI**—Social health insurance; **TB**–Tuberculosis; **UCT**—Unconditional cash transfers; **UHC**–Universal Health Care.

### Development of topic guides

Prior to all interviews, interview topic guides were developed and the questions were mapped against the study’s acceptability framework [29]. Questions were developed for all acceptability constructs, although for the constructs related to opportunity costs and self-efficacy, questions were limited to the post-intervention interviews. As this was a socioeconomic intervention not focused on behavior change, limited questions focused on the construct of self-efficacy. Each topic guide was field tested during two individual interviews and modifications were made to the topic guides to clarify the phrasing of questions. (See S2 File–Topic Guide)

### Sampling

Participants were purposively sampled and to ensure maximum variation and ensure representation of all implementation districts and provide gender balance [33]. The concept of information power was used to guide the sample size and it was determined that due to the sample specificity and use of the established TFA theory, high quality dialogue and an understanding of framework analysis, a moderate sample size was required to assess the study’s aim [34]. Participants included 14 beneficiaries (people enrolled in the intervention), five non-beneficiaries (community-members), 13 TB staff from the national-, provincial- and district-levels and two program staff, who were employed by non-government organizations conducting this research. (See Table 3) Participants were initially approached over the telephone and invited to an in-person interview. Thirty-eight participants were invited to participate and the positive response rate was 89%. Four participants who initially agreed to participate did not present for the interview due to scheduling conflicts.

**Table 3:** Summary of study participants.

Pre-intervention interviews	Individual interviews (19 total)		Focus group discussions (3 total)		Total Participants	
	M	F	M	F	M	F
Provincial-level TB staff	2	1	-	-	2	1
District-level TB doctors	3	1	-	-	3	1
National TB program staff	1	1	-	-	1	1
Community members/Non-beneficiaries	-	-	2	3	2	3
Pre-intervention participants sub-total	9		5		14	
**Post-intervention interviews**						
District-level TB doctors	2	2	-	-	2	2
CCT + SHI beneficiaries	1	1	3	2	4	3
UCT + SHI beneficiaries	1	1	3	2	4	3
Program staff	-	2	-	-	0	2
Post-intervention participants sub-total	10		10		20	
**Total number of participants**	**19**		**15**		**34**	

**CCT**—Conditional cash transfers; **UCT**—Unconditional cash transfers; **SHI**—Social health insurance.

### Participant characteristics

In the pre-intervention interviews, participants included three staff from the Ho Chi Minh City Provincial Lung Hospital, four from district-level TB facilities, and two from the national-level TB program. Five community members (non-beneficiaries) were interviewed. Post-intervention interviews focused on those who had distributed, received or otherwise helped administer the intervention. Four doctors who worked at DTUs participated, along with four beneficiaries (2 CCT+SHI, 2 UCT+SHI). Two FGDs were conducted with beneficiaries (1 CCT+SHI, 1 UCT+SHI) who had received cash transfers UHC+. Lastly, two program staff who had administered the intervention were interviewed by a researcher based in Hanoi who had no role in delivering the intervention.

### Research team characteristics

Five interviewers conducted the study; all were female. TTN and LN conducted 14 of 22 interviews and were employed as Project Managers at the Center for Development of Community Health Initiatives. All interviewers had either Bachelor’s or Master’s degrees in social work or public health, had a combined 15 years of health-related interview experience, and were trained in qualitative data collection methods by KSA before conducting the interviews. KSA and RF, who developed the topic guides, have been trained in oral history and qualitative research methods. There were no pre-existing relationships between the study participants and the interviewers for the pre-intervention interviews, but the district-level TB doctors and the beneficiaries of the intervention had previously interacted with the interviewers before the post-intervention interviews.

### Data collection

The interviews were conducted at DTUs, at the National Lung Hospital, the Ho Chi Minh City Provincial Lung Hospital or the study office. Two interviews required a follow-up phone call to make clarifications and to collect missing information. All interviews were conducted in Vietnamese. Audio recordings were made for each interview and the interviewer collected field notes. In all 34 interviews, the interviewer, an interviewer’s assistant and the participants were present. In one FGD, a young grandchild of a participant also attended. Individual interviews averaged 64 minutes, while FDGs averaged 126 minutes.

### Data processing, management and analysis

Audio recordings of interviews were transcribed. Transcriptions were checked for fidelity by one of the interviewers. Transcripts were anonymized by removing the participant’s name and the district, hospital, and/or department where they worked. Transcripts were translated into English and verified by study staff who are bilingual in Vietnamese and English. Discrepancies were discussed with TTN and edits were made to the final English transcripts.

Initial coding was independently undertaken by RF and KSA through a close reading of the text using open coding. The list of codes generated was grouped into categories to index reoccurring concepts and ideas within the data by RF and KSA, and a common coding framework was agreed upon. This resulted in a final list of seven categories and 48 codes. Definitions for each of the codes were written to ensure consistent usage. (See S3 File) Codes were then applied to the English transcripts in Dedoose version 8.3.35 (Los Angeles, CA: SocioCultural Research Consultants, LLC www.dedoose.com) by RF and TD. If there were any discrepancies in the application of codes, they discussed and agreed upon the final coding.

Following coding, a code application chart was produced within Dedoose which enabled RF to read all qualitative data indexed by interview and code. Matrices summarizing participants’ views by code were produced and included interviews in the rows, codes in the columns, and summaries in the cells. One primary construct from the TFA was assigned to each of the 48 codes, and in 15 instances, a secondary construct was defined. Thematic matrices consisting of code summaries, were made for each of the TFA acceptability constructs. Two additional matrices were generated to summarize perceptions of acceptable uses of cash and the process of distributing the cash to beneficiaries. (See S3 File)

RF and KSA read down and across the thematic framework matrices and highlighted similar and discordant views described by interview participants to generate the results. The results were developed into themes and subthemes. The quotes below illustrate the themes, including the time of interview (pre/post) to the intervention, interview number, sex (M/F) and category of participant.

## Results

**Fig 1:**
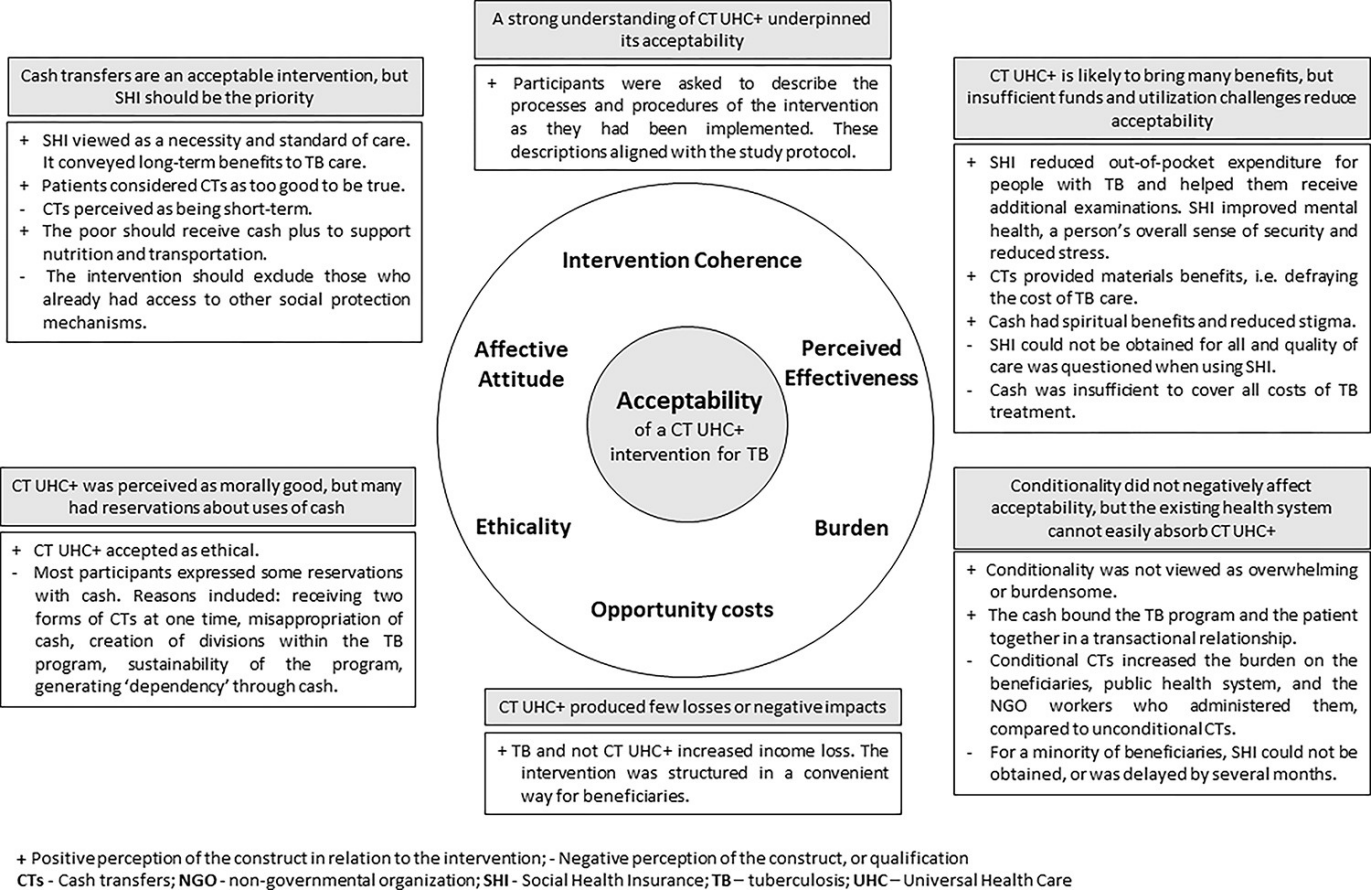
Summary of themes and subthemes arranged by interconnected acceptability constructs for a cash transfer UHC+ intervention for TB.

Six of the seven interrelated components of acceptability were identified within the qualitative data including: affective attitude, perceived effectiveness, ethicality, burden, intervention coherence and opportunity costs. The findings have been presented by themes and subthemes, grouped within the six acceptability constructs. (See Fig 1)

### Affective attitude

Participants across all categories described generally positive feelings about providing cash to people with TB. The cash transfers were seen as having short-term, immediate benefits for the people with TB, while SHI was perceived as having long-term benefits, since coverage would last for one year and cover morbidity beyond TB. The components in combination were mostly viewed as complementary, because it took time to register for SHI and the cash was needed most acutely in the first months of TB treatment. If either benefit was to be prioritized, then in the perspective of community members and the majority of TB doctors from the district- to national-level, SHI should be prioritized over cash.

Across all groups of participants, in both the pre-and post-intervention interviews, SHI was viewed as standard of care, a national priority, and a necessity. One doctor summed up this policy objective by saying, ‘The purchase of health insurance is something that every citizen must aim for, not only for TB patients (Pre-7-M, Provincial-level TB staff).’ While a beneficiary said, ‘We have to admit that health insurance is very necessary. For older people, disease can come at any time’ (Interview Post-11-F, CCT beneficiary). Reasons for not purchasing SHI prior to TB diagnosis included being unable to afford it for the entire household and low perceived risk of not having insurance. ‘I thought since I am still young, I don’t need it’ (Post-11-M, CCT beneficiary).

Post-intervention interviews about the cash transfers described them as unbelievable, and almost too good to be true. Participant 1 (Female): ‘I told my neighbor [about the cash transfer] but she didn’t believe that we received money. She said that treatment was free, but I said I also received money. She said I lied. Several times when I was given support [at my home], she was staring at us.’ Participant 2 (Male): ‘In my department at work, they don’t believe me’ (Post-12, UCT beneficiaries).

All participants were asked who should be eligible to receive a cash transfer intervention. Most participants believed that the support should be targeted towards the poor, with those in the most need prioritized first. Additional target groups viewed as deserving of financial support included the uninsured, internal migrants, those with chronic illnesses, the elderly, and those without official documentation, as they would be unable to obtain stable jobs. Several district-level doctors mentioned excluding those who already had access to other social protection mechanisms, such as those who receive monetary support through the Fund for Victims of Agent Orange.

Cash provision was deemed acceptable by every category of participant when used to support nutrition or food. Only two participants, both working for the TB program, were unconcerned about how cash was spent by people with TB. After using cash for nutritional support, TB staff and doctors tended to express positive feelings towards cash being used to pay for travel. However, people with TB tended to prioritize vitamin supplementation during TB care (See S3 File—Acceptable Uses of Cash).

### Perceived effectiveness

Participants described cash transfers as bringing material, moral, motivational and psychological benefits, which they often described as ‘spiritual’ benefits. Beneficiaries viewed cash as a way to defray the direct costs of treatment and direct, non-medical costs such as travel. Several doctors and program staff viewed them as a way to enable beneficiaries to purchase more expensive, nutritious foods such as fruit, allow them to buy medications not covered by the TB program, or to pay down debts.

In the opinions of a district and a provincial-level TB doctor, the cash allowed individuals with TB to rest and focus on recovery. Cash was identified as one component of a care model to improve treatment and reduce stigma.

‘The most important thing is the spiritual support; our [the intervention’s] support will make them feel that they are cared for and receive the additional care from other people. They feel moved and happy when they are cared for. …The support makes them feel that there is no discrimination against them. Because some people think that this [TB] is a dangerous and fatal disease… people will keep away from them. When we are with them… we sit with them and our action—our care for them through the cash support—is the way to reduce stigma.’ (Post-9-F, Program staff)

One beneficiary stated that the cash transfer, ‘helped me to understand and appreciate my life.’ (Post-11-F, CCT beneficiary) TB doctors and program staff also described cash as improving attitudes about TB treatment and helping beneficiaries, who were struggling through treatment, feel more content while seeking healthcare.

‘Cash transfers help our patients feel happy and we are happy when our patients are happy, because sometimes they say, “With this support, I will buy more food for my family, or I will buy this or that”. … [With the cash,] they pay more attention to their health; they buy tonics and take care of their health better. They confidently go for health examinations and spend more on themselves.’ (Post-10-F, Program staff)

While describing benefits of cash transfers, the specific amount of cash provided in the intervention was considered by all beneficiaries as being insufficient. All participants were asked how much money should be provided per month and the average amount was 2.5 million VND per month (~107 USD, OANDA.com), which was five times more than the amount provided by the intervention. Beneficiaries expressed gratitude for receiving any money and were hesitant to comment negatively on the support package, but they felt that the value of the cash transfer was insufficient to cover their food costs or additional medication. Some participants stated that any amount of cash was better than nothing, but one respondent made it clear that even though there was need, ‘Patients don’t dare to ask for more.’ (Interview Post-12-M, UCT beneficiary).

The primary utility of SHI for beneficiaries was seen as reducing out-of-pocket expenditure during TB treatment. Staff of the TB program felt that SHI was often undervalued by beneficiaries, since TB medication was largely provided free-of-charge through the NTP. But both beneficiaries and doctors identified coverage of costs for TB comorbidities as a specific benefit of SHI. Health insurance was viewed as expanding access to care, thus improving TB care, and reducing out-of-pocket expenditure. Doctors, program staff and beneficiaries described the ways SHI improved the beneficiary’s mental health, a person’s overall sense of security and reduced stress.

The benefits of SHI were described as allowing people with TB to focus on their care and recovery. ‘Mentally, if they get health insurance, they are not worried about their disease anymore and do not have to worry about treatment costs as they have health insurance as their amulet. They don’t have to worry and can focus on other things such as their economic situation and their family.’ (Pre-2-M, District-level TB doctor).

However, beneficiaries in the post-intervention interviews described SHI enrollment and utilization challenges. A small proportion of beneficiaries who were eligible for SHI enrollment, were unable to register for SHI since they did not possess the correct official documentation, or the registration process took more than 30 days. Additionally, when beneficiaries described their experiences using SHI when accessing auxiliary TB care and non-TB care, they felt that the care provided to those who paid out-of-pocket was superior to the care received when using SHI. Five UCT beneficiaries in a FGD believed that they received care from ‘junior doctors’ when using SHI, not more senior clinicians (Post-12, UCT beneficiaries). Negative perceptions of services provided under SHI were described. One beneficiary said, ‘Seeking health care with health insurance is not as pleasant as seeking health care without using it. It’s slower. Those who don’t use health insurance are prioritized.’ (Post-5-F, Beneficiary) These challenges limited beneficiaries’ belief that the intervention would support them during care or reduce their TB-related expenditure.

### Ethicality

Across all participant groups, cash transfers were viewed as aligned with their value systems, and perceived as morally beneficial. None of the participants opposed the distribution of cash outright. However, many expressed reservations with the provision of cash.

Several TB doctors were concerned with how the money would be spent. One said, ‘Money is the motivation for them to follow the treatment and we hope that the money will turn into food or something to improve their health.’ (Pre-7-M, Provincial-level TB staff)

Five categories of reservations were identified in the interviews. The first was that beneficiaries would obtain funding from another government agency along with the funds reserved for people with TB. The second was that cash could be misappropriated for lottery tickets, heroin, or other purposes not contributing to the alleviation of the socioeconomic burdens of TB. The third was that cash transfer had the potential to be socially divisive, create groups of beneficiaries and non-beneficiaries, cause conflict between doctors and patients, and increase the complaints fielded by the doctors. The fourth was that the existing funding sources could not sustain a cash transfer program and future stoppage of payments would disrupt treatment adherence. Lastly, TB doctors expressed concerns about ‘dependency.’ They were concerned that the cash would cause beneficiaries to become too reliant on the services of the TB program, causing them to disengage with their treatment and increase the workload of the DTU. ‘When we offer support, the patients shall become dependent. It means that while some patients understand and obey the treatment plan, the others rely on us as they think, “I do not care, it is covered by the program.”‘ (Post-2-F, District-level TB doctor)

Dependency was also a concern for people with TB. In a post-intervention FGD, beneficiaries suggested that instead of providing small monthly cash transfers, they would prefer access to credit. They saw their only options for obtaining credit outside of their personal networks as coming from the Capital Aid for Employment of the Poor Microfinance Institution or from predatory lending agencies. These funds were seen as difficult to obtain, while loans from the black-market had high interest rates, repayment plans that were difficult or impossible to meet, and the inability to repay loans from the black-market had dire consequences. Beneficiaries specifically suggested that they requested loans up to 10 million VND (~428 USD, OANDA.com), and they should be provided without requiring any collateral as security for the loan. This suggestion to provide credit instead of cash aligned strongly with their fear of dependency. Beneficiaries expressed an unease with receiving a gift of cash in case it made them indebted to the state, a non-governmental organization or another entity. Both TB doctors and beneficiaries had the same desire–for people with TB to remain independent, take accountability for their own treatment, not allow the cash transfer UHC+ intervention to replace their intrinsic motivation to get well, and help provide for the needs of their families.

### Burden

National, provincial and district level TB doctors often felt that conditions related to treatment adherence, such as following the treatment plan by taking medications according to schedule and attending follow-up clinic appointments should be required to receive cash. As one doctor put it, ‘We don’t give money for nothing.’ (Pre-3-F, Provincial-level TB doctor). The money was viewed as binding the patient and the provider in a financial relationship and helping people with TB understand the importance of treatment.

However, even without conditions, an UCT beneficiary described feeling a similar transactional relationship and experiencing acute feelings of shame when he was unable to adhere to his TB treatment plan. ‘I think everyone wants to take medicine to cure the disease. Now we receive the money but we don’t take the medicine and our disease is not cured. We go to the DTUs, they say this or that, we feel ashamed. I am ashamed of myself because I have not completed my mission. What is my task? Just to take the medicine, but I don’t finish it. It is not acceptable. The disease will not disappear.’ (Post-12-M, UCT beneficiary)

The specific conditionality of the intervention was not perceived as overly burdensome by any of the participants. Program staff who had assessed adherence to the conditions stated, ‘This is not complicated at all. This is the way to check whether the patients use all medicines or not. And it does not impact them much; we only request them to return once per month.’ (Post-9-F, Program staff).

For the health system, the added burden of monitoring the conditions was perceived to improve the quality of TB care at the DTU and to bolster TB treatment adherence among beneficiaries. Speaking about a CCT participant, one TB doctor noted:

‘He was eligible for the nutritional [cash transfer] and health insurance support from the program. After 2 months of treatment monitored by the DTU, he returned home for the Lunar New Year Festival. Then he stopped treatment and we only noticed this after he stopped for almost 2 months. This was detected thanks to the intervention as he also didn’t come to receive the support money. After checking all the documents, we found out that he had stopped treatment. The TB officer in the ward then contacted the patient to continue the treatment.’ (Post-2-F, District-level TB doctor)

Even with the perceived improvements in treatment adherence brought about by the conditions, CCTs were viewed by the majority of the district-level health staff as overly burdensome to monitor, outside their remit, and impossible to scale-up. One doctor in charge of a district where program staff distributed CCT made his opinion clear about the possibility of administering a CCT program in the future. ‘If there are one or two patients, it’s okay. But there are more than 50 patients. We don’t have enough time to check all [the conditions]. What nonsense! … The salary paid by the state does not cover this extra job, the structure of the health examination service does not include this work.’ (Post-3-M, District-level TB doctor)

Program staff also felt that CCTs took too much time, effort and cost to administer, compared to UCTs. ‘We have to travel a lot, from this place to that other place and we need time to talk and encourage the patients. It takes half an hour for some patients and even one hour for others… We are very busy, sometimes we have to travel from morning to afternoon.’ (Post-9-F, Program staff)

### Intervention coherence

In post-intervention interviews, study participants were asked to explain the processes, procedures and delivery schedule for SHI, CCTs and UCTs as they had been implemented. All participant types were able to describe the intervention components in detail, which indicated high intervention coherence within the cash transfer UHC+ intervention.

### Opportunity costs

Participants tended to describe an added burden, and not a loss or personal cost, from participating in the cash transfer UHC+ intervention. Study and TB staff did not feel that there were opportunity costs associated with participating in the intervention. For beneficiaries, the home- or DTU-based payment locations were found to be convenient, and integrated well into their existing travel plans for TB treatment. Even though they were specifically asked about the costs and potential negative impacts of the intervention, beneficiaries blamed TB disease and treatment for income loss, not the intervention.

## Discussion

Across six interconnected acceptability constructs, cash transfers were broadly accepted, for both CCT and UCT. SHI was perceived as having longer-term benefits, such as improving and expanding TB care and reducing out-of-pocket expenditure. From a health system perspective, if limited funding is available and only one benefit could be supported, SHI should be given priority since it is viewed as standard of care in Vietnam. However, the cash transfer was viewed as complementary to SHI and conveying material and ‘spiritual’ benefits to reduce stigma and discrimination during TB care; therefore, from a social protection perspective, there is support for cash transfer UHC+ interventions. Cash was viewed as a means to increase contact between the healthcare provider and the person with TB, improve the patient-provider partnership and help the person with TB recover a full sense of self.

However, the value of the cash transfer was insufficient to offset the full costs of TB care, and there were widespread concerns both about the ways the money could be spent and generating a sense of ‘dependency’ among beneficiaries. The conditionality of CCTs was not perceived negatively, but was described as a transaction between the person with TB and provider, whereby cash was exchanged for TB treatment adherence. However, UCT beneficiaries understood this transaction in a similar way to CCT beneficiaries, without the added burden of meeting the conditions to receive payment. While still acceptable, CCTs were perceived as being overly burdensome to administer by district-level doctors in the TB program and program staff.

A recent systematic review synthesizing qualitative evidence on experiences of cash transfers to improve health outcomes showed that cash transfers were perceived to produce positive personal and social outcomes, enhance empowerment, increase hope, and improve social cohesion. It also found that interactions with the cash transfer program impacted the acceptability of the intervention [35]. Cash transfer programs in other settings were acceptable when stakeholders held positive perceptions of the impact of a program, they promoted community participation and reduced social division [36–38]. This study supported these findings from the literature by showing that affective attitude and perceived effectiveness played a large role in the acceptability of a cash transfer UHC+ intervention, and that stakeholders worried about creating discontent among non-beneficiaries when providing the cash transfers.

The conditionality of cash transfers has been a longstanding subject of policy debate in terms of its effectiveness, acceptability, and ethicality [39–45]. Conditionality has been found to be acceptable when it is perceived as fair and as a proxy measurement for a positive outcome, such as achievement or merit. However, conditioning a cash transfer based on access to care and treatment adherence has been flagged as potentially impractical and unethical [46]. Questions regarding the ethicality of the conditionality of cash transfers persist, specifically in regards to respect for persons, fairness and unintended negative consequences. Ethicists have described conditionality as ‘ethical’ in the design phase of an intervention if it 1) increases the likelihood of bettering a health outcome; 2) takes into account risks and burden imposed; 3) assesses the willingness and resistance to meeting program conditions; 4) establishes conditions that are attainable; and 5) considers the indirect impacts that the conditionality may have on the beneficiaries [45]. This study reveals that during the design phase of the intervention, the conditionality of the CCT satisfied those ethical considerations. However, the relative gains brought about by the conditionality should be quantified. Future studies should investigate the role of conditionality on improving TB treatment outcomes, reducing catastrophic cost incurrence for TB-affected households, and the burden of administering conditions on the health system.

Within this study, cash transfers were found to be less of a priority for almost all participants than the provision of SHI. This is perhaps unsurprising from the health system’s perspective in Vietnam since SHI coverage is national policy and 90% of the general population has already been enrolled [16]. These findings may also align with the hesitancy expressed in other settings to apply for cash transfer benefits due to a distrust in government, or a social desirability bias to not appear ‘lazy’ [47–49].

While SHI was preferred over cash transfers, participants discussed barriers to SHI enrollment and utilization challenges. A prior study from Vietnam has shown that ‘money changing hands’ enhanced the contractual patient-provider relationship and perceived quality of care [50]. Nonetheless, utilization of SHI needs to be addressed in order to maximize its benefits, and truly achieve UHC. Additionally, data from a longitudinal patient cost survey conducted in Ho Chi Minh City showed that income loss and household expenditure for TB were highest during the intensive phase of drug-susceptible TB treatment, typically in the first two months [51]. This suggests that if SHI provision were to be prioritized over cash as a social protection mechanism for TB in Vietnam, there is still a need for a policy availing access to direct financial support during the first months of TB care for those who enter treatment uninsured.

Social protection interventions solely providing cash transfers in LMICs are unlikely to have adequate resources to provide a sufficient value to mitigate catastrophic costs from TB [52]. Cash transfers are generally perceived as necessary and helpful, but often only sufficient to cover some immediate needs and not always to achieve the outcomes of the program [35]. For this reason, cash transfer models combining multiple forms of social protection are required. In this study, acceptability of the cash transfer declined when it was perceived as insufficient to defray the costs of TB care. Beneficiaries requested access to other forms of social protection, such as low- or no-interest loans. Microcredit institutions have the potential to become self-sustaining, but they tend to target those who are able to pay back the loans, they have been shown to increase indebtedness, require infrastructure to track and seek repayment, and their success hinges on the management capacity of the microcredit institution [53]. While microcredit may improve general economic wellbeing, this type of intervention has not yet been proven to improve TB outcomes, and the challenges with designing and implementing a policy for TB-affected households remain significant, while protecting borrowers from predatory lenders [46].

Layering multiple forms of social protection and increasing access to national schemes for long-term sick leave and sickness insurance, should be considered in the design of programs to ensure that support is adequate. Within the Vietnamese context, this will require multisectoral collaboration with entities outside of the traditional health-care infrastructure including the Ministry of Labor Invalids and Social Affairs (MOLISA). In addition, there are often unaddressed psychosocial needs during TB care that require social support [54–60]. Social support during TB care has been shown to reduce stress and assist in the recovery process [61,62]. Although this intervention was not specifically designed as a ‘cash plus care’ model, providing tailored psychosocial support, the regular engagement with and counselling provided by program staff was viewed as a benefit and core component of the acceptability of the intervention [63].

The cash transfer UHC+ intervention evaluated in this study was designed to investigate support with similar components to those provided by the government’s PASTB fund. Vietnam is one of the first LMICs to create a nationally-available social protection fund specifically dedicated for people with drug-susceptible TB to reimburse the purchase of SHI and provide cash transfers [18]. PASTB aligns with the UHC goals of extending services to non-covered people and reducing cost sharing and fees [64]. This research on cash transfer acceptability can inform the design of social protection schemes distributed by PASTB and similar mechanisms in other countries. It indicates that it is broadly acceptable for SHI reimbursement to remain the priority of the PASTB fund. However, this study also pointed to the need for optimization of existing SHI services and additional staffing support for overburdened healthcare workers when delivering social protection. One potential way to complete this is through better integration of social workers within the health system and the scale-up of PASTB.

To monitor progress in achieving the WHO End TB Strategy target of ‘No TB affected families experiencing catastrophic costs due to TB’, the proportion of people with TB who experience catastrophic costs are published annually in WHO’s Global TB Report for selected countries, [3,65]. Now that baseline measurements have been established in some countries, research activities should focus on evaluating interventions to reduce or mitigate catastrophic costs [66].

## Methodological considerations

### Theoretical framework of acceptability

The TFA developed by Sekhon and colleagues is becoming widely cited, primarily because it is the first attempt to systematically develop a common understanding of the concept of acceptability [29]. A smaller number of studies have utilized the TFA in qualitative research to structure the presentation of results [67–69].

When designing topic guides and conducting interviews, all seven components of the TFA were included [29]. (See S2 File) However, within our qualitative data, four of the constructs (affective attitude, perceived effectiveness, ethicality and burden) provided the richest insights. Intervention coherence and opportunity costs underpinned the acceptability of the intervention and yielded expected insights.

In this study, all beneficiaries and district-level TB and program staff who implemented the intervention were able to accurately describe the services provided, the schedule and the conditions required to receive the CT. We believe this was facilitated by clear standard operating procedures, robust data systems, responsive implementers, and a strong understanding of the socioeconomic vulnerability for the population of people with TB. In this case, high intervention coherence undergirded the high acceptability. However, poor intervention coherence caused by a lack of understanding about the way an intervention has been designed to function, poor training, or a general misunderstanding of the theory of change underpinning an intervention, has great potential to reduce its acceptability. Our study did not find a high opportunity cost associated with participating with the intervention. This was mostly due to the design of the intervention, and it being integrated into existing care processes.

Researchers conducting qualitative research may find that not all of the TFA constructs are appropriate for all interventions, as was found during the development of the quantitative TFA Questionnaire [27]. The TFA defines self-efficacy as “the participants confidence that they could perform the behaviors to participate in the intervention.” In our study, this was the definition used to develop topic guides related to this construct, and it was felt from the outset that this construct was more applicable to behavior change interventions. Upon further reflection, additional questions could have been asked to better assess if the cash transfers UHC+ intervention improved beneficiaries’ beliefs that they could complete TB treatment according to programmatic guidelines. When utilizing the TFA and developing questions related to self-efficacy, researchers should consider specifying the behaviors required to participate in an intervention, or modify the definition of self-efficacy to the participants belief that they can perform the behaviors required to achieve the intervention’s intended outcome.

### Strengths and limitations

The study benefits from a high level of trustworthiness [70]. Utilizing the acceptability framework helped structure both the topic guide and the framework analysis, creating transparency and improving dependability. Purposive sampling for maximum variation with different stakeholders from beneficiaries to facilitators enables transferability.

A limitation of the paper is that cash transfer beneficiaries had previously engaged with program staff who conducted the interviews. This could potentially lead to social desirability bias in the qualitative responses. Other limitations include the fact that four components of the 32-item COREQ Checklist were not included in this study. The personal goals of the researchers were also not explained to study participants and their biases are not described in the manuscript. Furthermore, due to staffing and time constraints, transcripts were not provided to participants and they were not consulted about the findings.

## Conclusion

The question of acceptability is foundational for the development of interventions that build supportive systems for ending TB in Vietnam and in other LMICs. Cash transfers are one mechanism of social protection that has the potential to address the social consequences and determinants of TB. We conclude that this cash transfer UHC+ intervention which delivered both UCT and CCT was acceptable to beneficiaries, implementers and policy stakeholders in Vietnam. However, more research is needed to understand the impact of these cash transfers on health and economic outcomes and their cost effectiveness in order to create an enabling policy environment for the scale-up of similar interventions nationally and internationally.

## Supporting information

S1 File COREQ checklist. (PDF)

S2 File Topic guide. (DOCX)

S3 File Code definitions and matrices. (XLSX)
