# Supplementary material for: A qualitative assessment on the acceptability of providing cash transfers and social health insurance for tuberculosis-affected families in Ho Chi Minh City, Vietnam
Source: PLOS Glob Public Health. 2023 Dec 6;3(12):e0002439. doi: 10.1371/journal.pgph.0002439 (PMC10699628; doi:10.1371/journal.pgph.0002439)
Supplement: S2 File — (DOCX) [file pgph.0002439.s002.docx]

# Topic guide for the cash transfer UHC+ intervention– qualitative component

*Objective****:*** To explore the acceptability and appropriateness of Social Health Insurance (SHI) along with conditional and unconditional cash transfers, views and attitudes on the value, and different modalities from the participants’ perspective (both individuals enrolled in the study and those involved in implementing the study) at two time points. Further, to explore challenges associated with enrolling households on the SHI scheme from an operation and participant perspective.

*Study design*: Exploratory qualitative study with individual interviews and focus group discussions (FGDs).

*Study site:* Ho Chi Minh City (HCMC): Districts 8, Go Vap, Binh Chanh and Tan Binh. Employees of Viet Nam’s National TB Control Program will be interviewed in Hanoi.

*Participants*: Individual interviews with key informants will include both decision makers and implementers among public health officials and non-governmental organizational (NGO) staff. The FGDs will have three different groups (conditional, unconditional cash transfer beneficiaries and members from the community (non-beneficiaries) to explore the attitudes and perception toward the cash transfer (conditional or unconditional) and implementation of enrolling households on SHI. FGDs are also an appropriate method to help identify potential issues with the different modalities of the intervention and explore the most efficient method.

*Table 1:* *The estimated number of study participants, timing of interviews and type of methodology*

|  | **Individual Interviews** | **FGDs** | |
| --- | --- | --- | --- |
| *Timing: Before implementation of the intervention* | | | |
| Key informants (public health officials i.e. national, provincial and district TB officers & NGO staff) | 9 | | - |
| Community members (non-beneficiaries) |  | | 2-3 |
| *Timing: 3 months after implementation of the intervention* | | | |
| Key Informants | 2-3 | | - |
| Unconditional cash transfer beneficiaries | 2-3 | | 1 |
| Conditional cash transfer beneficiaries | 2-3 | | 1 |

*Individual Interviews:* The purpose of these interviews is to assess perceptions of the cash transfers from the perspective of a variety of stakeholders. The same topics above will be explored before the intervention is implemented however the addition of the feasibility of facilitating the transfer (additional time, process etc) will be explored after at least three months of implementation.

*Focus Group Discussions (FDGs):* The purpose of the FGDs with the different beneficiaries and community members is to learn about participant and community perceptions of cash transfer impacts on recipient households and on the overall community. We will explore positive and negative perceptions and concerns about the cash transfer program, the conditionality; perceptions of recipient behaviors regarding health seeking behaviors and use of the cash transfer; the community‘s perceptions of the fairness of targeting; and the existence of possible jealousy among non-beneficiary households. To further explore a concept from the FGDs – we will perform individual interviews with 4-6 of the participants.

*Data collection:* Data will be collected at two different time points (see table 1 above): before the intervention is implemented and at least three months after implementation. A second time point has been chosen at least 3 months after implementation, so participants have adequate experience distributing/receiving the cash, and understanding the challenges and impact of the social health insurance and cash transfer before the interview. Data collection in this manner will allow the research team to understand perceptions before the intervention is introduced but also actual barriers to implementation and unintentional consequences.

Theoretical framework of acceptability: based on Sekhon TFA model - there are 7 constructs (followed by their definition) to explore acceptability of an intervention. These topics will be explored from both the participants and program implementers’ perspective:

1. **Affective** **attitude** (how participants feel about the intervention)
2. **Burden** (perceived amount of effort that is required to participate in the intervention)
3. **Ethicality** (the extent to which the intervention has a good fit with the participants value system)
4. **Intervention Coherence** (the extent to which the participant understands the intervention and how it works)
5. **Opportunity costs** (the extent to which benefits, profits, or values must be given up to engage in the intervention)
6. **Perceived Effectiveness** (extent to which the intervention is perceived as likely to achieve its purpose)
7. **Self-efficacy** (the participants confidence that they can perform the behaviors required to participate in the intervention)

|  | **Pre-Intervention Interviews** | | **Post-Intervention Interviews** | |
| --- | --- | --- | --- | --- |
|  | **Key Informants** | **Community Members** | **Key Informants- NGO staff & District-level TB staff** | **Beneficiaries** |
| **1. Affective** **attitude** (how participants feel about the intervention) | How do you feel about people with TB receiving cash transfers & SHI? | How do you feel about people with TB receiving cash transfers & SHI? | How does it make you feel when you assist a person with TB to register his/her family on SHI? | How does it make you feel when you hear all people with TB will receive SHI? |
|  | How do you think cash transfers would be received in your community? | Combination of cash + SHI; Preference for cash vs. SHI | How does it make you feel when you give People with TB cash transfers? | How does it make you feel when you give People with TB cash transfers? |
|  | How should the cash be used? | How should the cash be used? | How should the cash be used? | How should the cash be used? How have you spent the cash transfers that you have received? |
|  | Acceptable source of social protection- NGOs vs. government | Acceptable source of social protection- NGOs vs. government |  |  |
| **2. Burden** (perceived amount of effort that is required to participate in the intervention) | Ideal process of providing cash: duration, best modality for cash transfer, best person to receive the cash transfer | Ideal process of providing cash: duration, best modality for cash transfer, best person to receive the cash transfer | Ideal process for providing cash: most convenient methods, duration of cash distribution, modality of cash transfer, best person to receive the cash transfer, and suggested changes in implementation | Ideal process for receiving cash: most convenient methods, modality of cash transfer, duration of cash distribution, best person to receive the cash transfer, and suggested changes in implementation |
|  | - | Other government sources of support |  | Experience with other sources of social protection from the government |
|  |  |  | Process of enrolling people with TB on SHI: convenience, challenges, differences in geography | Experience enrolling (or not enrolling) on SHI: convenience, ease, challenges, procedures, |
|  |  |  |  | Barriers to meeting the conditions (Conditional Cash Transfer recipients only) |
|  |  |  | Package implementation questions: time requirements, streamlining processes, enrollment criteria, scaling intervention |  |
| **3. Ethicality** (the extent to which the intervention has a good fit with the participants value system) | Who should receive the cash transfer/SHI? (i.e. should all people with TB? Just the poor? universal vs conditions. What is the eligibility criteria? | Who should receive the cash transfer/SHI? (i.e. should all people with TB? Just the poor? universal vs conditions. What is the eligibility criteria? | Who should receive the cash transfer/SHI? (i.e. should all people with TB? Just the poor? universal vs conditions. What is the eligibility criteria? |  |
|  | Conditionality- What kind of conditions should a person with TB have to meet in order to receive a cash transfer?  Appropriateness/fairness of the conditions to receive cash. | Conditionality- What kind of conditions should a person with TB have to meet in order to receive a cash transfer?  Appropriateness/fairness of the conditions to receive cash. | Conditionality- how do you feel about the conditions attached to providing the conditional cash transfer?  Appropriateness/fairness of the conditions to receive cash. | Describe your experience in meeting these conditions. How do you feel about the conditions attached to providing the conditional cash transfer? (Conditional Cash Transfer recipients only) |
|  | Reservations about providing cash to people with TB | Reservations about providing cash to people with TB | Reservations about providing cash to people with TB |  |
|  | Who should decide how the cash is spent? | Who should decide how the cash is spent? |  | How did you and/or your family decide how the money was spent? How is money handled in your household? |
| **4. Intervention Coherence** (the extent to which the participant understands the intervention and how it works) |  | What is SHI in Viet Nam? What is the difference between a cash payment and SHI? | Explain the process to distribute the cash transfers & enroll people with TB on SHI | Explain the process to receive the SHI & cash transfers. |
|  | Why would people with TB be enrolled in the social health insurance (SHI) scheme? | Why would people with TB be enrolled in the social health insurance (SHI) scheme? |  | Describe the conditions you need to meet/do in order to receive the cash transfer? (Conditional Cash Transfer recipients only) |
|  | Why would people with TB receive a cash payment? | Why would people with TB receive a cash payment? |  |  |
|  | - | Do people with TB require both the cash payment and SHI? | What do you feel is the optimal way to support people with TB? | What other types of support do people with TB need? |
| **5. Opportunity costs** (the extent to which benefits, profits, or values must be given up to engage in the intervention) | - |  | How did SHI enrollment impact people with TB in a negative way? | Experience enrolling on SHI: challenges, procedures, job loss.  Have you incurred additional costs/expenses associated with SHI?  Describe any negative consequences of having social health insurance |
|  |  |  | What are the key differences between administering the conditional and unconditional cash transfers? What would you change about the provision of cash transfers? (Only for NGO staff) |  |
|  |  |  | How has your work been impacted by implementing this intervention? | Have you incurred additional costs/expenses associated with cash transfers? |
| **6. Perceived Effectiveness** (extent to which the intervention is perceived as likely to achieve its purpose) | Perceived impact of SHI; Adequacy/inadequacy of SHI; uses of SHI | Perceived impact of SHI; Adequacy/inadequacy of SHI; uses of SHI | How were people with TB affected by SHI? | How were you able to use the social health insurance for TB-related expenses? What did SHI do for your family during your treatment? |
|  | Influence of the cash transfer on their health seeking behavior (i.e. easier to attend appointments) and treatment adherence (i.e. easier to take medication) | Influence of the cash transfer on their health seeking behavior (i.e. easier to attend appointments) and treatment adherence (i.e. easier to take medication) | How have you seen people with TB be affected by the cash transfer? | Describe how the cash transfer has affected your health-related behavior |
|  | Adequacy of the monetary transfer value | Adequacy of the monetary transfer value | Adequacy of the monetary transfer value | Adequacy of the monetary transfer value. Was it enough to alleviate the costs of TB? Describe your need for cash at the beginning, middle and end of treatment. |
| **7. Self-efficacy** (the participants confidence that they can perform the behaviors required to participate in the intervention) | - |  |  | How will you maintain enrollment on social health insurance next year? How prepared do you feel to re-enroll on SHI? |

# **Topic Guides**

**Before implementation:**

## *Participant: Key informants (public health officials i.e. national, provincial and district TB)*

*The objective of this study is to understand the participant’s’ perceptions of providing a combination of social health insurance and cash transfers to people with TB.*

*Begin with icebreaker: Ask the key informant about their role within or supporting the TB program.*

**Affective** **attitude** (how participants feel about the intervention)

- How do you feel about people with TB and their households receiving social health insurance?
  - *Probe:* Understand the rationale behind the answer
  - *Probe*: NGOs vs. government funding purchasing SHI for People with TB and their households?
- How do you feel about people with TB receiving cash transfers?
  - *Probe*: Understand the rationale behind the answer
  - *Probe*: From which sources should cash transfers be given?
- How do you think a project that provides cash to people with TB would be received in your community?
- What should the recipient (of the cash transfer) be able to do with the money?
  - *Prompt*: Would it be on individual specific items, for the household, related to treatment?
  - *Probe:* What should the recipient of the cash transfer not be allowed to do with the money?

**Burden** (perceived amount of effort that is required to participate in the intervention)

- For how long should this amount of cash be given to people with TB? Why?
- If people with TB are to receive cash, when should they receive the transfers? Motivate why those times.
- Who in the household should receive cash transfers? Why?
  - *Prompt*: just the person on TB treatment, the head of the household, multiple people

**Ethicality** (the extent to which the intervention has a good fit with the participants’ value system)

- Who should receive the cash transfer? Why?
  - *Probe*: All people with TB? Just the poor? All people?
- What kind of conditions should a person with TB have to meet in order to receive a cash transfer? Why?
  - *Probe:* If there may be difficulties in meeting the conditions they mention. If yes – how can they be overcome?
  - *Probe:* Reservations about providing cash transfers
- Who should decide how the cash is spent? (head of household, person with TB, spouse, etc.)

**Intervention Coherence** (the extent to which the participant understands the intervention and how it works)

- Why would people with TB be enrolled in the social health insurance (SHI) scheme?
  - *Probe: H*ow do you envision this helping a person with TB?
- Why would people with TB receive a cash payment?
  - *Probe*: How could this help a person with TB?
  - *Prompt:* Access to treatment, afford nutritious food to eat during treatment

**Perceived Effectiveness** (extent to which the intervention is perceived as likely to achieve its purpose)

- What will be the impact of SHI on a TB-affected person’s life?
- How would the cash transfer affect the participants’ behavior?
  - *Prompt*: How would it change health seeking behaviors (i.e. how and when they access health services (appointments) and treatment (adherence to medication)
- We are trying to understand the amount of money that you think people with TB would require for one month. This is the amount of cash after the person with TB received social health insurance for the year. What do you feel would be an appropriate and fair value for a cash transfer to a person with TB for…. (*interviewer should read through categories 1-5*)

| Category: | Amounts: | Why this amount? |
| --- | --- | --- |
| 1. Food |  |  |
| 2. Transport |  |  |
| 3. Living costs (rent, utilities, clothes, etc.) |  |  |
| 4. Medical costs |  |  |
| 5. Any additional expenses |  | What is included in this category? Why this amount? |
| **Total** | *Interviewer should add the amounts in categories 1-5 and ask…* |  |

- Is the total of __________ VND a value that you believe to be an appropriate and fair amount that should be provided to a person with TB per month?
  - *Probe*: If they feel that the total value is too high, then probe to understand why it is too high and see where they would make adjustments to the categories

**Is there anything else you would like to tell us?**

**Before implementation:**

## *Participant: Community members (non-beneficiaries)*

*The objective of this study is to understand the participant’s’ perceptions of providing a combination of social health insurance and cash transfers to people with TB.*

*Begin with icebreaker: Ask the community members to describe what they know about TB. Ask if they know anyone who has had TB.*

**Intervention Coherence** (the extent to which the participant understands the intervention and how it works)

- What is Social Health Insurance in Viet Nam?
- What is the difference between a cash payment and SHI?
- Why would people with TB be enrolled in the social health insurance (SHI) scheme?
- Why would people with TB receive a cash payment?
  - *Probe*: How could this help a person with TB?
  - *Prompt:* Access to treatment, afford nutritious food to eat during treatment

**Affective** **attitude** (how participants feel about the intervention)

- How do you feel about people with TB and their households receiving social health insurance?
  - *Probe:* Understand the rationale behind the answer
  - *Probe*: NGOs vs. government funding purchasing SHI for people with TB and their households?
- How do you feel about people with TB receiving cash transfers?
  - *Probe*: Understand the rationale behind the answer
  - *Probe*: From which sources should cash transfers be given?
  - *Prompt*: the role of government, NGOs, research studies, religious groups, individual benefactors
- In your opinion, does a person with TB need both a cash payment and SHI? Ask to motivate answer.
- What should the recipient (of the cash transfer) be able to do with the money?
  - *Prompt*: Would it be on individual specific items, for the household, related to treatment?
  - *Prompt:* What should the recipient of the cash transfer not be allowed to do with the money?

**Ethicality** (the extent to which the intervention has a good fit with the participants’ value system)

- Who should receive the cash transfer?
  - *Probe*: Should all people with TB? Just the poor? All people?
- What kind of conditions should a person with TB have to meet in order to receive a cash transfer? Why?
  - *Probe*: If there may be difficulties in meeting the conditions they mention. If yes – how can they be overcome?
  - *Probe:* Reservations about providing cash transfers
- Who should decide how the cash is spent? (head of household, person with TB, spouse, etc.)

**Burden** (perceived amount of effort that is required to participate in the intervention)

- For how long should this amount of cash be given to people with TB? Why?
- If People with TB are to receive cash transfers, when should they receive them? Motivate why those times.
- Who in the household should receive cash transfers? Why?
  - *Prompt*: Just the person on TB treatment, the head of the household, multiple people
- Can you describe other government programs to alleviate financial constraints during sickness?
  - *Prompt*: Describe the types of programs available for poor households in HCMC
  - *Probe*: If these programs are used. If not – why? (barriers)

**Perceived Effectiveness** (extent to which the intervention is perceived as likely to achieve its purpose)

- What will be the impact of SHI on a TB affected person’s life?
- How would the cash transfer affect the participants’ behavior?
  - *Prompt*: How would it change health seeking behaviors (i.e. how and when they access health services (appointments) and treatment (adherence to medication)
- We are trying to understand the amount of money that you think People with TB would require for one month. This is the amount of cash after the person with TB received social health insurance for the year. What do you feel would be an appropriate and fair value for a cash transfer to a person with TB for…. (interviewer should read through categories 1-5)

| Category: | Amounts: | Why this amount? |
| --- | --- | --- |
| 1. Food |  |  |
| 2. Transport |  |  |
| 3. Living costs (rent, utilities, clothes, etc.) |  |  |
| 4. Medical costs |  |  |
| 5. Any additional expenses |  | What is included in this category? Why this amount? |
| **Total** | *Interviewer should add the amounts in categories 1-5 and ask…* |  |

- Is the total of __________ VND a value that you believe to be an appropriate and fair amount that should be provided to a person with TB per month?
  - *Probe* If they feel that the total value is too high, then probe to understand why it is too high and see where they would make adjustments to the categories

**Is there anything else you would like to tell us?**

## *Participant: Key Informants – NGO Staff & District-level TB staff*

**Social health insurance**

- Please describe the process of enrolling people with TB on social health insurance
  - How do you feel about this process? → Probe for convenience, challenges, ease, differences between districts
  - What would you change about the provision of social health insurance?
- At the beginning of the pilot project, the project was unable to register several eligible people with TB on SHI because of their lack of paperwork or difficulties with their paperwork. Looking back, how should the NGO have approached social health insurance enrollment differently?
- For those who received it, how have you seen people with TB be affected by their enrollment on SHI?
  - →How did the intervention impact them in a positive way?
  - →How did it impact them in a negative way? → Prompt – time away from work, loss of privacy, lost sense of independence
- How does it make you feel when you assist a person with TB to register his/her family on SHI?

**Cash transfers**

- What do you feel is the optimal way to support people with TB?
  - If not cash, then how else do people with TB need support?
- Please explain the process to distribute the cash transfers
  - How do you feel about this process? → Probe for convenience, ease
  - Can you describe the most ideal process to distribute the cash? And why is it most ideal? (i.e. electronically or a receiving it at a different location/time)
- What are the key differences between administering the conditional and unconditional cash transfers?
  - → Probe – efficiency of both kinds of transfer and unintentional consequences assisted with either of type
  - What would you change about the provision of cash transfers?
- Who should be eligible to receive the cash transfer? Why?
  - Probe: All people with TB? Just the poor? All people?
- Now that you have experience administering the cash transfers, what do you feel would be an appropriate and fair value for a cash transfer to a person with TB for…. (interviewer should read through categories 1-5)

| Category: | Amounts: | Why this amount? |
| --- | --- | --- |
| 1. Food |  |  |
| 2. Transport |  |  |
| 3. Living costs (rent, utilities, clothes, etc.) |  |  |
| 4. Medical costs |  |  |
| 5. Any additional expenses |  | What is included in this category? Why this amount? |
| **Total** | *Interviewer should add the amounts in categories 1-5 and ask…* |  |

- Is the total of __________ VND a value that you believe to be an appropriate and fair amount that should be provided to a person with TB per month?
  - Probe: If they feel that the total value is too high, then probe to understand why it is too high and see where they would make adjustments to the categories
- Now that we are implementing this intervention, how do you feel about the conditions attached to providing the conditional cash transfer?
  - →Probe - What kind of conditions are fair? Why?
- What should the recipient (of the cash transfer) be able to do with the money?
  - Prompt: Would it be on individual specific items, for the household, related to treatment?
  - Probe: What should the recipient of the cash transfer not be allowed to do with the money?
- For how long should this amount of cash be given to people with TB? Why?
- If people with TB are to receive cash, when should they receive the transfers? Motivate why those times.
- Who do you feel should receive the cash transfer in the family?
  - → Probe - understand the role of the person within the family
  - If different from the current person, ask why
- How have you seen people with TB be affected by the cash transfer?
  - → Probe for both positive and negative
  - → Probe to see if the amount was adequate for the needs of the People with TB
- How has your work been impacted by implementing this intervention?
  - → Probe for both positive and negative
- How does it make you feel when you give people with TB cash transfers?

**Implementation considerations**

- How do you feel about the time required of you to participate in this project? Prompt: how can the work be streamlined?
- How do you feel about your participation in this project?
- How do you feel about the enrollment criteria of the project? If you could change it, how would you change it?
- How do you feel about the overall implementation of the project?
- If this intervention was going to be scaled-up to more districts and more cities, what would you change about it?

**Is there anything else you would like to tell us?**

## *Participant: Unconditional or conditional cash transfer beneficiaries*

**Poverty alleviation schemes:**

- Describe your experience with other government programs to alleviate financial constraints?
  - *Prompt: Vocational training, reduction in school fees, loans for small businesses, overseas worker program*
  - → Probe if these programs are used. If not – why?
  - → Probe if these programs are used, ease of use, challenges, barriers

**Social health insurance questions**

- Before you had TB, could you describe your prior experience with obtaining or attempting to obtain social health insurance?
  - → Probe convenience, ease, challenges, cost, procedures, job loss, etc.
  - → Probe (if applicable) what are the reasons why you don’t insurance now?
- (If applicable) For those who did *not* have insurance -> Can you describe the reasons why you did not have health insurance?
  - → Probe affordability, privacy, coverage of SHI, family issues
- (If applicable) Please explain the process of receiving social health insurance. *Prompt: from whom, when, how long did it take and where did they receive the SHI.*
  - How do you feel about this process? → Probe for convenience, ease, challenges
  - What would you change about the provision of social health insurance?
- (If applicable) Were you able to use the social health insurance for TB-related expenses?
  - If yes, how did it work?
  - If no, why were you unable to use it? →Probe- need to use SHI during TB treatment, knowledge of procedures to use it, decided to not use it
- What did SHI do for your family during your treatment?
- Have you incurred additional costs/expenses associated with SHI? If yes – why?
  - How did you pay for those additional costs/expenditures?
- Describe any negative consequences of having social health insurance. *Prompt*: less ability to choose services or specific medications? Down payment at the hospital?
- How does it make you feel when you hear all People with TB will receive SHI?
- (If applicable) How will you maintain enrollment on social health insurance next year? How prepared do you feel to re-enroll on SHI?

**Cash transfer questions**

- Please explain the process to receive the cash transfers. *Prompt: from who, when and where do they receive the money.*
  - How do you feel about this process?
    - Probe for convenience, ease, challenges
  - If you could receive them in any way, how would you like to receive the payments?
    - *Prompt: bank transfer, ViMomo, Moca, VietPay, cash,*
    - *Prompt: every week, every other week, every month*
  - Thinking about the cash payments over the last few months, what would you change about how we gave you these cash transfers?
- Describe the conditions you need to meet/do in order to receive the cash transfer?
  - Describe your experience in meeting these conditions
  - How do you feel about the conditions attached to receiving the conditional cash transfer?
  - Probe for any barriers they may have encountered in trying to meet/adhering to the conditions
- Have you incurred additional costs/expenses associated with cash transfers? If yes – why?
  - How did you pay for those additional costs/expenditures?
- How have you spent the cash transfers that you have received?
  - *Prompt*: individual specific items, for the household, related to treatment?
- How did you and/or your family decide how the money was spent?
  - *Prompt*: who made the decision?
- In general, how is money handled in your household?
  - Probe: Who decides how the cash transfer will be spent for the household?
  - Who in the household should receive cash transfers? Why?
  - Probe - just the person on TB treatment, the head of the household, multiple people
- How do you feel about the amount of cash that was provided?
  - *Prompt*: Was it enough to alleviate the costs of TB?
- Describe your need for cash at the beginning, middle and end of treatment.
  - *Prompt*: did you require the same amount of cash each month of treatment or did you require more during specific timepoints?
- Based on your experience, if this money was to be given to another person with TB, for how many months do you feel they would need financial support?
- What other types of support do People with TB need?
  - Probe: education, counselling, vocational training
  - Probe: from whom? The government, NGOs, community organizations, DTU
- Describe how the cash transfer has affected your health-related behavior. *Prompt*: In regards to your general health, how and when you access health services (appointments), and treatment (adherence to medication)
- How does it make you feel when you hear all People with TB will receive cash transfers?

**Is there anything else you would like to tell us?**
